# Supplementary figures and images for: Interactions at the Oviposition Scar: Molecular and Metabolic Insights into Elaeagnus angustifolia’s Resistance Response to Anoplophora glabripennis
Source: Int J Mol Sci. 2024 Aug 31;25(17):9504. doi: 10.3390/ijms25179504 (PMC11395401; doi:10.3390/ijms25179504)

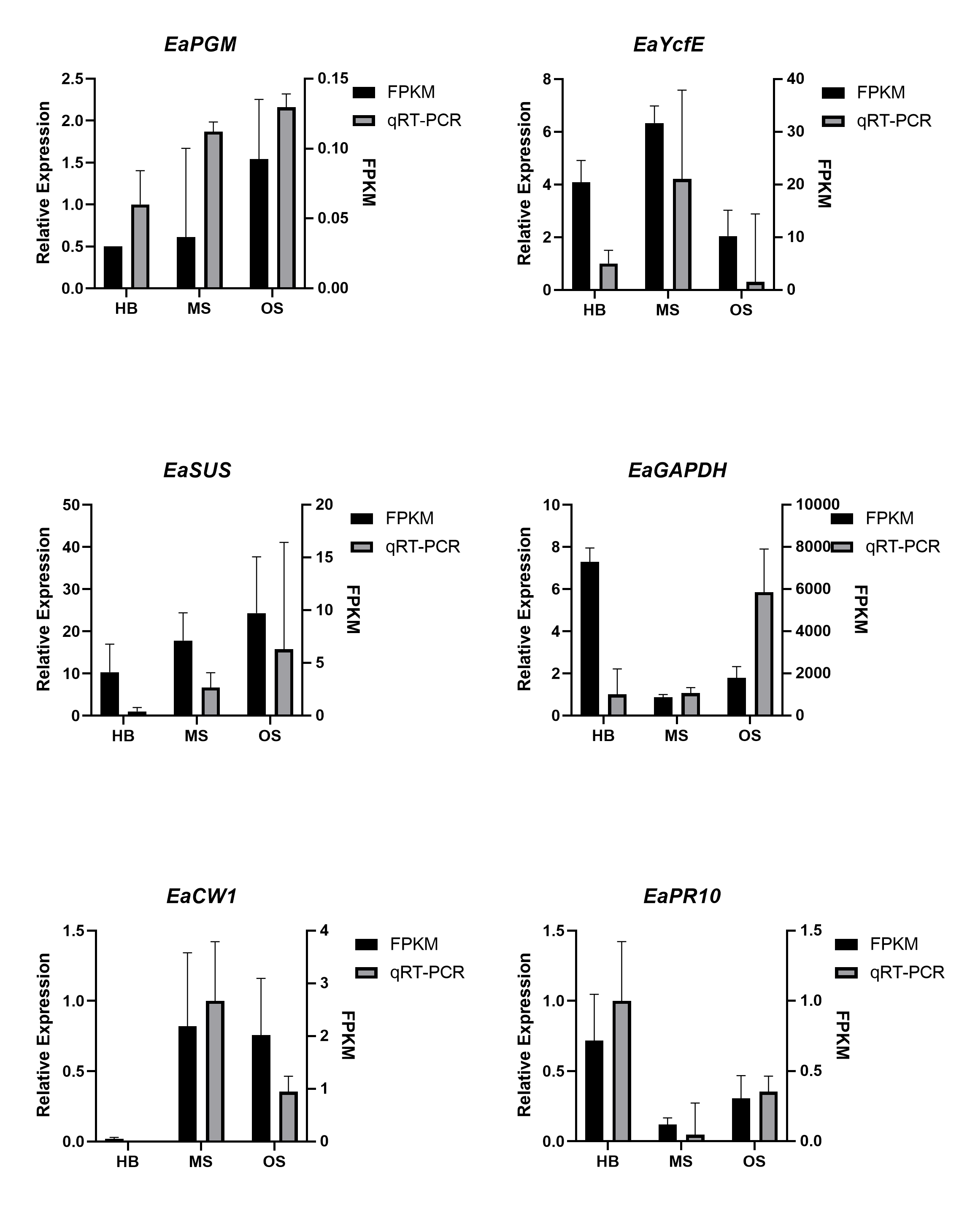

Supplement: Supplementary file 1 [file ijms-25-09504-s001.zip › ijms-3140295-supplementary/Figure S2-qPCR (2).png]

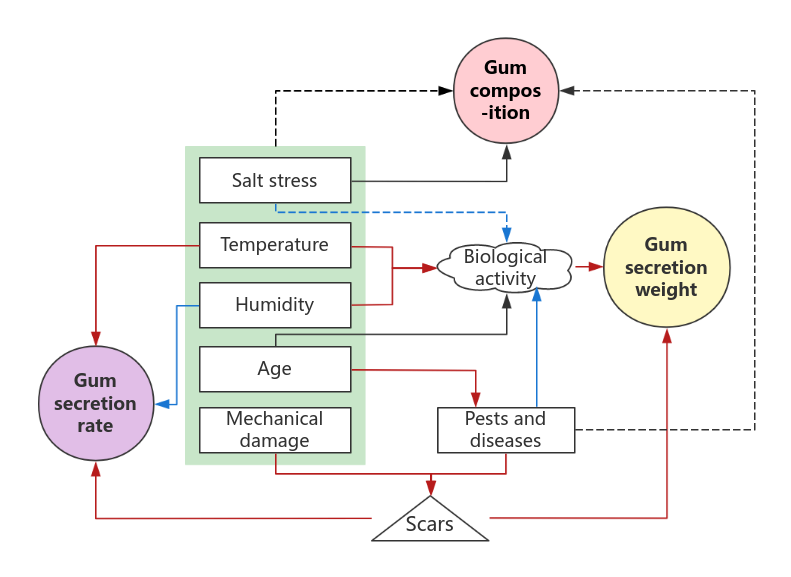

Supplement: Supplementary file 1 [file ijms-25-09504-s001.zip › ijms-3140295-supplementary/Figure S3.png]
